# Developing stem cell-based therapies for neural repair

**DOI:** 10.3389/fncel.2013.00198

**Published:** 2013-11-05

**Authors:** Clare L. Parish, Lachlan H. Thompson

**Affiliations:** Florey Institute of Neuroscience and Mental Health, The University of MelbourneParkville, VIC, Australia

**Keywords:** stem cells, neural repair, transplantation, drug disovery, disease modelling, embryonic stem cells, pluripotent stem cells, nerve tissue proteins

Current pharmacotherapies and surgical intervention provide limited benefit in the treatment of neural injuries or halting disease progression. Significant advances in the area of stem cell biology in the last decade has lead to a special emphasis on the success of stem cell-based applications for the diagnosis and treatment of neurological conditions. The properties of stem cells render them appropriate for endogenous repair, disease modeling, high-throughput drug screening and development as well as neural transplantation procedures. Such applications will aide in increasing our knowledge and developing treatments for neurodegenerative disorders such as Parkinson's disease and Huntington's diseases as well as neural traumas including ischemic brain damage and traumatic brain injury. This Frontiers Research topic incorporates contributions from the general field of stem cell biology, with a particular emphasis on utilizing these cells to develop new therapies for neural repair. Related articles deal with issues including: promoting endogenous neurogenesis, breakthroughs in stem cell differentiation methodologies, using pluripotent and neural stem cells for transplantation, patient derived stem cells for disease modeling and using stem cells for drug discovery.

The first 3 papers focus on understanding and subsequently exploiting, endogenous neurogenesis in order to enhance neural repair. Within the adult mammalian brain, neurogenesis is largely restricted to two sites; the dentate gyrus of the hippocampus and the subventricular zone adjacent to the lateral ventricle. In order to “harness” these cells for repair it will be vital to understand the mechanisms regulating their proliferation, migration, differentiation and survival. Sui et al. (2013) examines the role of the neuropeptide cholecystokinin (CCK) and its receptor (CCK1R) in this context. It is now well recognized that existing rates of neurogenesis in the adult brain are insufficient to replace neurons lost to neurodegeneration or trauma. Taylor et al. (2013) provide an overview of the field of hippocampal neurogenesis, the consequences of ageing on these populations, as well as changes in mood-related disorders and touches on efforts to activate this largely quiescent cell population. Christie and Turnley (2013) provide a broader review of extrinsic and intrinsic factors that have been shown to regulate adult neurogenesis, and highlight those that have already shown evidence of preclinical efficacy.

The generation of stem cell lines provides valuable tools for disease modeling and drug development. Blastocyst screening for genetic disorders such as Huntington's disease and Down's syndrome have enabled the generation of embryonic stem cells carrying these mutations. In more recent years, inducible pluripotent stem (iPS) cell technology has allowed stem cell lines to be generated from somatic cells of patients suffering from a number of degenerative conditions including Parkinson's disease and motor neuron disease. Added to this has been the ability to generate neural stem cell lines from other sources such as olfactory ensheathing cells of patients. Combined, such cell lines provide new tools to examine the pathological events that lead to disease onset and progression as well as development and testing of novel drug targets. Here, Niclis et al. (2013) examines 2 Huntington disease derived ESC lines. Whilst they demonstrate these lines show many normal properties, including gene expression, that are typically dysregulated in the disease, subtle changes that may shed further insight into the disease are observed. An article by Mackay-Sim (2013) provides a review on the use of stem cells for high throughput, and high content screening of large chemical libraries in efforts to identify novel drug targets. Such targets can then be utilized for drug development and again tested on patient-derived stem cell lines in culture.

One of the most widely anticipated applications for pluripotent stem cells is their utilization in neural transplantation procedures. This will require robust and appropriate fate restriction into more specialized lineages and their survival and appropriate functional integration into existing host circuitry after transplantation. Kirkeby et al. (2013) contribute a detailed methodological paper on the generating regionally specified neurons, by way of controlling dorso-ventral and rostrocaudal patterning of stem cells. Arber and Li (2013) provide a more focused review of the literature on understanding cortical interneuron development and the progress in protocols to yield interneuron subpopulations from pluripotent stem cells for the purpose of transplantation into models of epilepsy. In an original research article, Yoshikawa et al. (2013) examine the potential of 2 commonly used anti-convulsant drugs to improve the differentiation and survival of iPSC-derived dopaminergic neurons *in vitro* and *in vivo*, with this knowledge having implications for improved grafting approaches for treatment of Parkinson's disease. Garcia et al. (2013) highlight advancement in tools and technology that enable us to more accurately assess the integration of transplanted neurons in the brain. Finally, Broughton et al. (2013) provide a review on the exogenous “chaperone” benefits that stem cell grafts may provide in neural repair. The focus of their review is on amnion cells; highlighting the non-tumorogenic, non-immunogenic and trophic benefits of an alternative stem cell source.

Collectively these works highlight the rapidly progressing stem cell field and draw attention to the current and on-going potential these cells have for improving our understanding of the healthy and diseased nervous system and their capacity to promote neural repair.
